# Reversible cerebral vasoconstriction syndrome in psychiatric settings: Context‐dependent diagnostic bias and consultation‐liaison psychiatry practice

**DOI:** 10.1002/pcn5.70309

**Published:** 2026-02-27

**Authors:** Kyohei Otani, Nobuyasu Imbe, Ryota Shindo, Shigekazu Kitamura

**Affiliations:** ^1^ Department of Psychiatry Kakogawa Central City Hospital Kakogawa Hyogo Japan; ^2^ Department of Neurology Konan Medical Center Kobe Hyogo Japan; ^3^ Department of Neurology Hakuhokai Kakogawa Hospital Kakogawa Hyogo Japan

**Keywords:** bipolar disorder, consultation‐liaison psychiatry, context‐dependent diagnostic bias, COVID‐19, reversible cerebral vasoconstriction syndrome, thunderclap headache

## Abstract

**Background:**

Reversible cerebral vasoconstriction syndrome (RCVS) is a potentially life‐threatening neurological condition characterized by thunderclap headaches and multifocal cerebral arterial vasoconstriction resolving within 3 months. Context‐dependent diagnostic bias in psychiatric settings may delay recognition of life‐threatening conditions including RCVS.

**Case Presentation:**

A 52‐year‐old Japanese woman with bipolar II disorder stable on lithium developed recurrent thunderclap headaches 9 days after mild COVID‐19 infection. Headaches were consistently triggered by hot showers, reached maximal intensity within seconds, and were described as the worst headache of her life. Initial neurosurgical evaluation included non‐contrast head CT but attributed symptoms to tension‐type headache without vascular imaging. The patient, dissatisfied with this explanation, sought re‐evaluation through our psychiatry outpatient clinic. Psychiatric consultation identified characteristic RCVS features, prompting urgent referral to a headache specialist. Magnetic resonance angiography on Day 31 revealed multifocal segmental vasoconstriction confirming RCVS. Calcium channel blocker treatment led to complete symptom resolution with radiological resolution confirmed at Day 100.

**Conclusion:**

This case illustrates how context‐dependent diagnostic bias can dangerously delay RCVS recognition in psychiatric settings. Thunderclap headache warrants immediate neuroimaging regardless of psychiatric comorbidity. Psychiatric consultation enabled appropriate diagnosis through collaborative evaluation with specialist neurology, underscoring the essential role of consultation‐liaison psychiatry at the medical–psychiatric interface.

## Graphical abstract

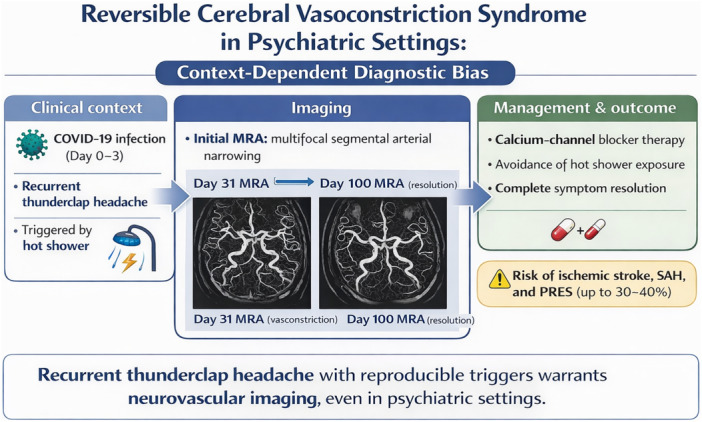

## BACKGROUND

Reversible cerebral vasoconstriction syndrome (RCVS) is a neurological emergency characterized by sudden‐onset thunderclap headaches and reversible multifocal cerebral arterial vasoconstriction.^1^ RCVS diagnosis requires the following: (1) acute severe headache with or without neurological deficits; (2) multifocal segmental vasoconstriction on cerebral angiography; and (3) documented reversibility within 3 months.^2^ Cases may be classified as definite RCVS when all criteria are met, or probable RCVS when reversibility confirmation is pending.^2^ Despite increasing neurological recognition, RCVS may be underrecognized in psychiatric settings where severe headache presentations face diagnostic challenges including symptom attribution to psychiatric illness or medication effects.^3^, ^4^

Thunderclap headache—the hallmark RCVS feature—reaches maximum intensity within 60 s, often described as “the worst headache of life.”^5^, ^6^, ^7^ However, this distinctive presentation may not trigger an appropriate neurological evaluation in patients with psychiatric diagnoses. Context‐dependent diagnostic biases—whereby known psychiatric illness shapes clinical interpretation of physical symptoms—can result in dangerous delays in recognizing life‐threatening conditions requiring immediate neurovascular imaging.^8^, ^9^, ^10^

RCVS has particular relevance for consultation‐liaison (C‐L) psychiatry. Prior literature has documented RCVS associated with psychotropic medications, highlighting medication management challenges and the critical role of psychiatry–neurology collaboration.^11^ Emerging evidence suggests that psychiatric patients—particularly those with bipolar disorder—may harbor specific cerebrovascular vulnerabilities including impaired cerebrovascular reactivity, chronic inflammation, and endothelial dysfunction.^12^, ^13^, ^14^, ^15^ SARS‐CoV‐2 infection has been temporally associated with RCVS onset, potentially through endothelial injury and systemic inflammation, though causality remains to be established.^8^, ^9^, ^16^

**Figure 1:**
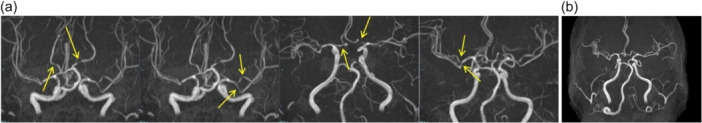
Magnetic resonance angiography (MRA) demonstrating reversible cerebral vasoconstriction syndrome. (A) MRA on Day 31 post‐COVID‐19 infection onset showing multifocal segmental vasoconstriction (arrows) affecting bilateral posterior cerebral arteries (P1 segments), bilateral anterior cerebral arteries (A1 segments), distal left middle cerebral artery (M1 segment), and right middle cerebral artery, with a characteristic “string of beads” appearance. (B) Follow‐up MRA on Day 100 demonstrating complete resolution of vasoconstriction with normalized vascular caliber across all previously affected territories, confirming definitive reversible cerebral vasoconstriction syndrome diagnosis.

We present a case of RCVS in a patient with bipolar II disorder with temporal association to COVID‐19 infection, in which context‐dependent diagnostic bias initially delayed diagnosis, and where C‐L psychiatry played a central role in recognition and appropriate referral (Figure 1).

## CASE PRESENTATION

A 52‐year‐old Japanese woman presented to our psychiatry outpatient clinic with severe recurrent headaches following COVID‐19 infection. She had a 23‐year history of bipolar II disorder, stable on lithium carbonate 600 mg daily (therapeutic serum levels 0.6–0.8 mEq/L). She had never received serotonergic antidepressants. Additional history included episodic migraine without aura (2–3 times yearly, responsive to non‐steroidal anti‐inflammatory medications), moderate alcohol consumption (36–48 g ethanol daily), and no history of illicit substance use. A comprehensive autoimmune evaluation 4 months prior had been entirely negative.

She developed mild COVID‐19 confirmed by PCR testing, with peak fever of 37.8°C and mild respiratory symptoms. She managed at home without hospitalization or antiviral medications. Lithium continued unchanged throughout the infection. She reported no psychiatric symptom exacerbation.

On Day 9 post‐COVID‐19 symptom onset, while taking a hot shower, she experienced sudden‐onset explosive headache reaching maximum intensity within 10–20 s—bilateral occipital, rated 10/10, described as “the worst headache of my life,” distinctly different from her typical migraine. Multiple similar episodes occurred over subsequent weeks, all consistently triggered by hot showering or bathing. Between episodes, she experienced a persistent lower grade background headache. Neurological examination revealed no focal deficits.

Initial neurosurgical evaluation on Day 12 included non‐contrast head CT showing no acute abnormalities. The presentation was attributed to tension‐type headache, without further neurovascular imaging such as MRI/MRA, despite the characteristic thunderclap onset and hot‐water trigger. This exemplifies context‐dependent diagnostic bias: awareness of the patient's psychiatric history may have contributed to an initial interpretation favoring a benign etiology, creating a dangerous delay in evaluation of a potentially life‐threatening condition.^10^ The patient was advised to use analgesics and manage stress.

Dissatisfied with this explanation and experiencing unchanged headache characteristics, the patient sought re‐evaluation at her regular psychiatry outpatient appointment on Day 26. This dynamic—a patient with a known psychiatric disorder bypassing the initial consultant and turning to her psychiatrist for a second opinion on a neurological complaint—reflects a common and important C‐L challenge. The treating psychiatrist recognized the characteristic RCVS features (thunderclap onset, reproducible hot‐water trigger, and recent COVID‐19 infection) and, noting that the case exceeded his headache expertise, directly contacted the co‐author (S.K.), a certified headache specialist, for urgent collaborative evaluation. The patient was informed that her symptoms were consistent with RCVS, a neurological condition unrelated to her psychiatric illness, and that specialist neurological assessment was needed. This explanation—validating her symptom concern and clearly distinguishing neurological from psychiatric etiology—was received with evident relief. An urgent neurological referral was arranged for neurovascular imaging.

MRI on Day 31 revealed no acute infarction, hemorrhage, subarachnoid hemorrhage, or posterior reversible encephalopathy syndrome (PRES). MRA demonstrated multifocal segmental vasoconstriction: bilateral posterior cerebral arteries (P1 segments), bilateral anterior cerebral arteries (A1 segments), distal left middle cerebral artery (M1 segment), and right middle cerebral artery. The characteristic “string of beads” appearance^17^, ^18^ confirmed RCVS diagnosis. Per Ducros (2012) criteria,^2^ this case was classified as definite RCVS, with documented vasoconstriction on MRA and subsequent complete radiological resolution. Laboratory evaluation showed no significant abnormalities. Lithium level was therapeutic at 0.7 mEq/L.

Treatment was initiated immediately. Given the urgency of RCVS—with risks of ischemic stroke, intracerebral hemorrhage, and PRES during the acute phase^19^—verapamil was started at 120 mg daily and titrated to 240 mg daily over 1 week with blood pressure monitoring. Lomerizine hydrochloride 10 mg daily was added. Trigger avoidance counseling included strict avoidance of hot water exposure and strenuous exertion. Lithium 600 mg daily continued unchanged given its lack of vasoconstrictive effects and importance for mood stability. Blood pressure was maintained at systolic 100–140 mmHg.

Thunderclap episodes ceased within 1 week of verapamil initiation. Follow‐up MRA on Day 100 demonstrated complete vasoconstriction resolution across all territories, confirming definitive RCVS diagnosis.^20^ Verapamil was tapered and discontinued at 3 months. At 6‐month follow‐up, she remained headache‐free, maintained euthymic mood on lithium monotherapy, and reported no neurological sequelae.

## DISCUSSION AND CONCLUSIONS

This case illustrates how context‐dependent diagnostic bias can critically delay RCVS recognition in psychiatric settings. The primary lesson is not RCVS itself, but the structural vulnerability in clinical reasoning that affects patients with psychiatric comorbidities: known psychiatric diagnosis may unconsciously—or consciously—redirect clinical attention away from serious neurological presentations. In this case, the patient's thunderclap headache with a typical trigger was initially evaluated with non‐contrast head CT, but interpreted as tension‐type headache without further neurovascular imaging, despite guidelines recommending vascular imaging for thunderclap headache.^10^ When psychiatrists maintain vigilance for medical illness in their patients, they may uniquely serve as a safety net that catches diagnostic errors made at other interfaces of care.

The C‐L dynamics in this case warrant explicit discussion, as they represent the operational core of C‐L psychiatry. The patient's return to her trusted psychiatrist after an unsatisfying neurosurgical consultation reflects the therapeutic alliance inherent in long‐term psychiatric care—an alliance that can be leveraged to identify medical emergencies. Critically, the role of the C‐L psychiatrist here was not to override the neurosurgeon's judgment, but to recognize a pattern inconsistent with the prior assessment and facilitate appropriate specialist re‐evaluation. Direct collaboration with the co‐author headache specialist enabled efficient, non‐adversarial resolution: the neurosurgeon's initial assessment was not publicly challenged, but the patient was redirected through established professional channels toward correct diagnosis. This approach—working within the hierarchy of care while prioritizing patient safety—represents optimal C‐L practice.

RCVS is characterized by sudden‐onset severe headaches and reversible multifocal cerebral arterial vasoconstriction predominantly affecting women in their fourth‐fifth decades.^21^, ^22^ The clinical and radiological spectrum ranges from uncomplicated presentations to severe complications including ischemic stroke (6%–39%), intracerebral hemorrhage (20%), PRES (9%–38%), and subarachnoid hemorrhage (22%–34%).^17^, ^19^ Neurovascular imaging should be performed urgently in any patient presenting with thunderclap headache: while some practitioners advocate imaging within 24 h of thunderclap onset, the risk of stroke and hemorrhage during the acute vasoconstriction phase supports the most expeditious evaluation feasible.^18^, ^23^ In our case, the 19‐day delay between symptom onset and MRA (Days 9–31) represents a clinically significant window during which complications could have occurred. Known triggers include vasoactive substances (serotonergic antidepressants, sympathomimetics), pregnancy, physical exertion, Valsalva maneuvers, and hot water exposure.^23^, ^24^

Differentiating RCVS from migraine is a critical clinical challenge.^2^, ^7^, ^25^ RCVS produces a thunderclap onset (maximum intensity within 60 s) versus a gradual migraine onset; extreme intensity (9–10/10) versus moderate‐severe; specific reproducible triggers (hot water, exertion, and Valsalva) versus variable migraine triggers; and multifocal vasoconstriction on vascular imaging versus normal vasculature in migraine.^26^ Our patient's prior migraine history initially complicated the assessment, but the thunderclap quality, severity, and consistent hot‐shower trigger clearly distinguished RCVS. Any thunderclap headache warrants immediate neurovascular imaging regardless of migraine history.^10^

Regarding the COVID‐19 association, this case demonstrates a temporal relationship between SARS‐CoV‐2 infection and RCVS onset, consistent with prior reports of COVID‐19‐associated RCVS.^8^, ^9^, ^16^ However, the presence of a clearly established RCVS trigger (hot shower exposure) and a recognized background factor (migraine history) necessitates caution in attributing causality to COVID‐19. The relationship in this case is best described as temporal association rather than causation: COVID‐19 may have contributed through endothelial injury or systemic inflammation,^8^, ^9^ but was one of multiple potentially predisposing factors alongside bipolar disorder–related cerebrovascular vulnerability,^12^, ^13^, ^14^, ^15^, ^27^, ^28^ migraine background, and alcohol use.

RCVS management centers on trigger avoidance, calcium channel blockers (verapamil 240–480 mg daily or nimodipine), and blood pressure management.^29^, ^30^ In psychiatric populations, serotonergic antidepressants should be avoided during acute RCVS given vasoconstrictive potential,^11^, ^31^, ^32^ while mood stabilizers including lithium can generally be continued. Recurrence risk of 3%–6% necessitates ongoing trigger avoidance and clinical vigilance.^20^ In our case, psychiatry–neurology collaboration not only enabled correct diagnosis but optimized psychotropic management throughout.

Key clinical pearls for C‐L psychiatry include the following: (1) thunderclap headache requires immediate neurovascular imaging regardless of psychiatric history; (2) specific reproducible triggers—particularly hot water exposure—strongly suggest RCVS; (3) the therapeutic alliance in psychiatric care can identify medical emergencies missed elsewhere; and (4) C‐L psychiatrists can serve as a critical safety net at the medical–psychiatric interface, facilitating correct diagnosis through specialist collaboration rather than adversarial challenge of prior assessments. This case underscores that maintaining vigilance for physical illness in psychiatric patients is not merely good practice—it can be life‐saving. The C‐L approach described in this case reflects the institutional C‐L framework at our center, including structured liaison nursing involvement previously described.^33^

## AUTHOR CONTRIBUTIONS

Kyohei Otani conceptualized the study, provided direct patient care, conducted literature review, and drafted the manuscript. Nobuyasu Imbe and Ryota Shindo contributed to data curation. Shigekazu Kitamura provided neurological and headache specialist consultation, interpreted neuroimaging studies, and revised the manuscript. All authors read and approved the final manuscript.

## CONFLICT OF INTEREST STATEMENT

The authors declare no conflicts of interest.

## ETHICS APPROVAL STATEMENT

This case report was conducted in accordance with the Declaration of Helsinki. Written informed consent was obtained from the patient for publication of this case report and accompanying images.

## PATIENT CONSENT STATEMENT

Written consent for case reports was obtained from the patient.

## CLINICAL TRIAL REGISTRATION

N/A.

## Data Availability

The data that support the findings of this study are available on request from the corresponding author. The data are not publicly available due to privacy or ethical restrictions. All data generated or analyzed during this study are included in this published article.
